# Toxin-like Peptides from the Bacterial Cultures Derived from Gut Microbiome Infected by SARS-CoV-2—New Data for a Possible Role in the Long COVID Pattern

**DOI:** 10.3390/biomedicines11010087

**Published:** 2022-12-29

**Authors:** Carlo Brogna, Simone Cristoni, Barbara Brogna, Domenico Rocco Bisaccia, Giuliano Marino, Valentina Viduto, Luigi Montano, Marina Piscopo

**Affiliations:** 1 Department of Research, Craniomed Group Facility Srl., 20091 Bresso, Italy; 2 ISB—Ion Source & Biotechnologies Srl., 20091 Bresso, Italy; 3 Department of Radiology, Moscati Hospital, Contrada Amoretta, 83100 Avellino, Italy; 4 Marsanconsulting Srl. Public Health Company, Via dei Fiorentini, 80133 Napoli, Italy; 5 Long COVID-19 Foundation, Garforth, Leeds LS25 1NB, UK; 6 Andrology Unit and Service of Life Style Medicine in Uro-Andrology, Local Health Authority (ASL), 84124 Salerno, Italy; 7 Department of Biology, University of Naples Federico II, 80126 Napoli, Italy

**Keywords:** COVID-19, toxin-like peptides, bacteriophage behavior, SARS-CoV-2, long COVID, gut microbiome

## Abstract

It has been 3 years since the beginning of the SARS-CoV-2 outbreak, however it is as yet little known how to care for the acute COVID-19 and long COVID patients. COVID-19 clinical manifestations are of both pulmonary and extra-pulmonary types. Extra-pulmonary ones include extreme tiredness (fatigue), shortness of breath, muscle aches, hyposmia, dysgeusia, and other neurological manifestations. In other autoimmune diseases, such as Parkinson’s disease (PD) or Alzheimer’s Disease (AD), it is well known that role of acetylcholine is crucial in olfactory dysfunction. We have already observed the presence of toxin-like peptides in plasma, urine, and faecal samples from COVID-19 patients, which are very similar to molecules known to alter acetylcholine signaling. After observing the production of these peptides in bacterial cultures, we have performed additional proteomics analyses to better understand their behavior and reported the extended data from our latest in vitro experiment. It seems that the gut microbiome continues to produce toxin-like peptides also after the decrease of RNA SARS-CoV-2 viral load at molecular tests. These toxicological interactions between the gut/human microbiome bacteria and the virus suggest a new scenario in the study of the clinical symptoms in long COVID and also in acute COVID-19 patients. It is discussed that in the bacteriophage similar behavior, the presence of toxins produced by bacteria continuously after viral aggression can be blocked using an appropriate combination of certain drugs.

## 1. Introduction

There has been a worldwide attempt to study SARS-CoV-2 virus for the past three years. The clinical aspects of COVID-19 disease, studies of the virus, and existing knowledge in the area of virology allowed many researchers to make various hypotheses on the underlying mechanisms driving the symptoms of the acute phase and of the long COVID; however, there is no common understanding on what causes these conditions and their treatment modalities. The probable origin of SARS-CoV-2, the fact that it seems to have as a close relative the bat coronavirus RATG13 [1], and that there is a divergence between the two coronaviruses, at least in the region binding domain (RBD) site of the virus major surface protein (spike protein) with the eukaryotic cellular region of the ACE2 receptor [2], do not seem to be enough. It seems that other unknown mechanisms could play a different role in the clinical picture of the neurological manifestations of the patients affected by the acute phase of COVID-19 or by Long COVID. One of the first Chinese studies described the neurological symptoms in a cohort of 214 patients. Of these patients, 36.4% had neurological manifestations, both of the central nervous system (CNS) and peripheral nervous system (PNS). Symptoms reported were dizziness, headache, impaired consciousness, acute cerebrovascular disease, ataxia, seizures, altered taste and smell, vision problems, nerve pain, and skeletal muscle injury [3]. In addition to this finding, another review also reports cerebral venous (sinus) thrombosis, epilepsy, meningitis, encephalitis, meningoencephalitis, Gullain–Barrè syndrome (GBS), Miller Fisher syndrome (MFS), acute myelitis and reversible posterior encephalopathy syndrome (PRES) [4,5]. Furthermore, in children, even if the current literature reports a low manifestation of the severe acute phase, it is possible to observe important neurological symptoms [6]. Regarding long COVID conditions, Premraj et al. [7] reported a statistical analysis of 1458 articles. The prevalence of post-COVID-19 neurological symptoms were observed: fatigue, brain fog, memory issues, attention disorder, myalgia, anosmia, dysgeusia, and headache, while neuropsychiatric conditions observed are sleep disturbances, anxiety, and depression. Some neurological symptoms such as anosmia or dysgeusia, or others are not only present in COVID-19 patients but are also described in other diseases like Parkinson’s (PD) [8,9] or Alzheimer’s (AD) [10]. It is noted that one important pathway implicated in these neurological disorders is the cholinergic system [11], and a possible role of this mechanism has also been observed in COVID-19 patients [12,13,14], and some authors have observed a decrease in Butyrylcholinesterase (BChE, BuChE), a pseudocholinesterase, implicated in the hydrolysis of many different choline-based esters, along with Acetylcholinesterase (AChE), in COVID-19 patients [15,16]. These authors [16] observed how the outcome of hospitalized cases correlated with low levels of these enzymes. They also observed how there is a correlation between these enzyme levels and the C-reactive protein (PCR) of the patients. It should be considered that these enzymes, AChE and BChE, are known in the literature to be important in choline reuptake and acetylcholine sequestration and degradation. The parasympathetic system is involved in the pathology of COVID-19, and the clinic described increasingly indicates its marked connection with the cholinergic system [12,13,14]. These mechanisms are similar to those observed in the clinical of toxicological manifestation [17,18]. Depending on the metabolites or toxicological peptides, an agonist effect on nicotinic and muscarinic receptors or saturation of AChE and BChE enzymes can be observed, resulting in hyperactivation of cholinergic signaling or blockade [17,18]. On the other hand, many papers show the connection between PD [19,20] or AD [20,21], and the gut microbiome. They observed how the microbiota and the composition of the bacterial population change in these diseases, in contrast to the healthy population. In general, it has been noted that bacteria produce toxins [22,23], and with regard to other coronavirus it has been observed that host cells can produce peptides able to inhibit the binding between viral particles such as the Spike (S) protein and the infected cell’s surface, and that the mechanism of action appears to interfere with its folding and prevent entry [24,25]. At the same time, a category of compounds better defined as antimicrobial peptides (AMPs) is known to be present in nature and to have antiviral properties [26,27]. They are usually cationic peptide molecules (in the range 10–60 amino acids) secreted to contrast microbes (bacteria, fungi, small parasites or viruses), but examples of anionic ones, due to abundance of aspartic and glutamic amino acids, have been reported as well [26]. AMPs can be produced by eukaryotic cells such as mammalian and insect ones (they are called “defense oligopeptides” [27]), but also by microorganisms such as bacteria [27]. Some AMPs show specific antiviral action, such as those against human immunodeficiency virus (HIV). Examples of collections of natural antimicrobial peptides are also available, such as the antimicrobial peptide database 3 (APD3) [28]. AMPs with antiviral action usually show a mechanism able to prevent viruses from binding to cells or interfering with viral replication mechanisms [29]. Observations show that AMPs against viruses [30] can be diversified into peptides derived from the heptad repeat 1 (HR1), heptad repeat 2 (HR2), or region binding domain (RBD) subunits of the spike protein [31]. These peptides can also be derived from other AMP peptides or derived from nonstructural proteins [31]. 

In our previous paper [32], we described the presence of bacterial-derived toxin-like peptides, present in plasma and urine, and faecal matter of COVID-19 patients despite the healthy control. The toxin-like peptides (P) observed had sequences similar to proteins known for their toxicological effect. The toxin-like peptides (P) that had higher quantification were those with conotoxin-like sequences (characterized by a particular amino acid sequence of four C-C-CC disulfide bridges), phospholipases, particularly, A2, phosphodiesterase, zinc-metalloproteinase, bradykinin-like. We also conducted the tests during the healing phases and found a very low expression rate. In a second work [33], we observed how Spike protein (S-recombinant 2019-nCoV S1 + S2 ECD protein- Sigma-Merck, St. Louis, MO, USA, cat. SAB5700592), and toxin-like peptides (P) at non-cytotoxic concentrations differentially disrupt the expression of some neuron-, glia-, and NSC-related genes critical during brain development.

On the other hand, in a previous paper, Petrillo et al. [34], have observed that in addition to the increase of viral RNA and the genesis of many mutations in the same bacterial cultures, some antibiotics tested determined an arrest of RNA replication. The purpose of the present analysis is to evaluate, again in vitro, the change in toxin metabolism (P-toxin-like peptides), within the same bacterial cultures, at the interval of 30 days and using the same antibiotics as in the previous paper. In the present work, it is aimed to integrate those data on the effect of the antibiotic on peptides (P) production in the bacterial cultures, derivate from faecal matter of COVID-19-positive patients to oropharyngeal nasal swab, where is present and increased SARS-CoV-2, despite to bacteria cultures derivate from the faecal matter of healthy persons. The study involves in the first part (material and methods and data results) the analysis of toxin-like peptides (P) production in bacterial cultures derived from faecal matter of sick patients, for COVID-19, up to 30 days. In the discussion, we emphasize the production of these molecules up to 30 days. The data show a decrease in the production of toxin-like peptides (P) with the introduction of certain antibiotics in vitro and suggest a possible role of the microbiome in perpetuating the long COVID phase of the disease.

## 2. Materials and Methods

A summary of the previous experiments and associated data can be found in [32,34]. The experimental design was reported by the authors [34].

In brief:

1. Culturing samples described in Petrillo et al. [34]: samples called A are the cultures of stool bacteria from COVID-19 patients; samples called B_(A+)_ are the cultures of stool bacteria from healthy people but contaminated with the supernatant from samples A; samples called C are the cultures of bacteria collected and grown after centrifuge of samples A and removal of the supernatant. Samples neg-B are the cultures of stool bacteria of healthy people that are the negative control. Moreover, an increase of RNA viral load up to day 30 of cultures in samples A and samples B_(A+)_, and how some antibiotics determine the decrease of viral RNA load in the cultures, was reported; in particular see Table 1. In addition, on aliquots of these cultures, the proteomic exams with the matrix-assisted laser desorption/ionization-time of flight (MALDI-TOF) technique and the surface-activated chemical-ionization (SACI) approach [34,35,36,37,38,39] were performed, as just described in [32], searching the unique new molecules that we have previously found in the plasma and urine of COVID-19 patients. The bacteria culture controls, derived from healthy persons, were negative for the increase of RNA viral load as previously described [34] and also for toxin-like peptides presence now reported. All patients gave their consent in accordance with Italian legislation.
2. Mass spectrometry data acquisition at different time points (beginning of culturing, after 7, 14, 21, 30 days) by means of Cloud ion mobility mass spectrometry (CIMS) coupled with surface-Electrospray-NIST-activated chemical ionization (SANS), followed by Surface Activated Chemical Ionization—Electrospray—NIST Bayesian model search (SANIST-CIMS) against the complete ‘Uni-Prot KB set of manually revised venom proteins and toxins’ [40] mixed with a subset of non-venom proteins and toxins from UniProt KB to give statistical significance to the results for the presence of proteins with potentially toxic effects.
3. Repetition of mass spectrometry data acquisition in the 18 aliquots derived from sample B_(A+)_ at day 21, where antibiotic tests were performed and consisting in the addition of a specific molecule (each of the following: metronidazole, clindamycin, lincomycin, piperacillin+tazobactam, vancomycin, amoxicillin, ampicillin, cefixime, ceftriaxone, meropenem, rifaximin, azithromycin, erythromycin, gentamicin, ciprofloxacin, colistin, levofloxacin, and teicoplanin), for detail see Table 1, previously described in [34].
4. Spectral counting [41] was performed in every aliquot, considering the toxin-like peptides abundance respect the culture-negative from SARS-CoV-2 derived from healthy patients. Spectral counting is a semiquantitative mass spectrometry approach for defining the abundance of the molecules under study. The spectral counting parameter was obtained using the exponentially modified protein abundance index (emPAI) [42] approach corrected by a nonparametric normalization index.
5. In order to verify the reproducibility of our results, the whole experiment was repeated three times independently.

## 3. New Data Results

**Figure 1:**
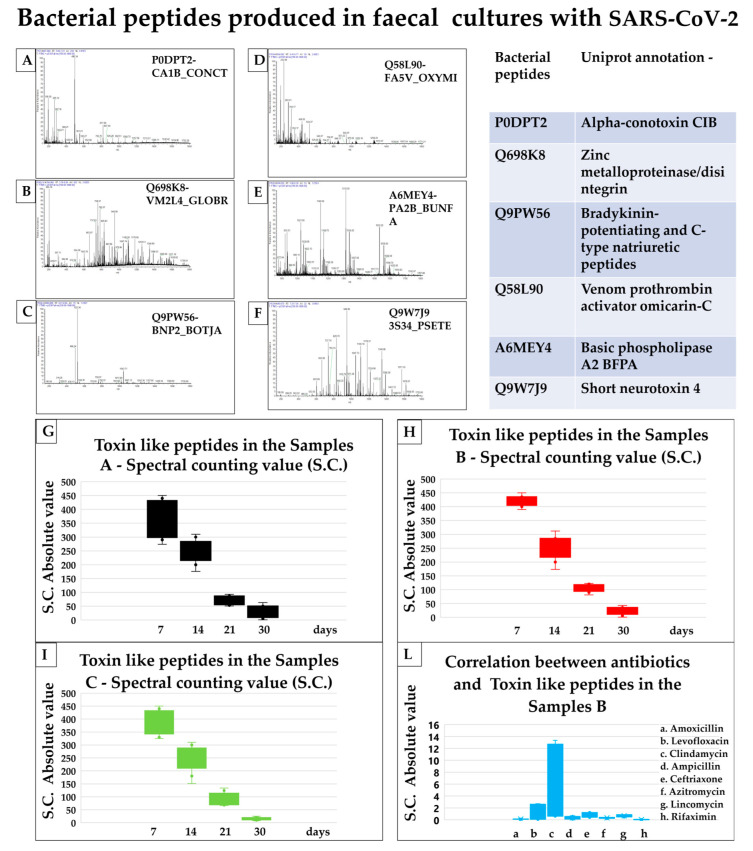

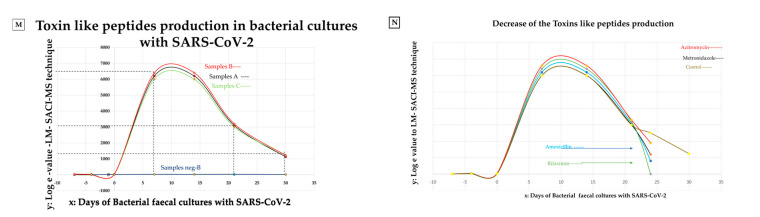
Toxin-like peptides data analysis. Panel (**A**–**F**): most commonly found toxin-like peptides (P). Panels (**G**–**I**,**L**,**M**) the increase of toxin-like peptides over time and the use of antibiotics. (Panels** A–F**): charge ratio mass spectra of the toxins-like peptides produced in faecal bacterial culture with SARS-CoV-2. Uniprot annotation ID. (Panel** A**): P0DPT2, alfa conotoxin-like peptides; (Panel** B**): Q698K8, zinc metalloproteinase/disintegrin; (Panel** C**): Q9PW56, Bradykinin- potentiating and C-type natriuretic peptides; (Panel** D**): Q58L90, venom prothrombin activator omicarin-C; (Panel** E**): basic phospholipase A2 BFBA; (Panel** F**): Q9W7J9, short neurotoxin 4. (Panels **G**–**I**,**L**): Spectral counting toxin-like peptides value to LC-SACI-CIMS technique. The values of the experimental repetitions at 7, 14, 21, and 30 days are represented by the box plots. The value of toxicological production in samples C (Panel** I**) is similar to those in samples A (Panel** G**) and B (Panel** H**), also in the absence of viral SARS-CoV-2 load. (Panel** L**): The amoxicillin and rifaximin stop the toxicological production versus other antibiotics in the bacterial cultures with SARS-CoV-2. (Panel** M**): High Log e value, LC-SACI-CIMS ion mobility technique, of toxin-like peptides (P) presence in samples A, B, and C and low Log e value of toxins in neg-B samples. (Panel** N**): Introducing four types of different antibiotics, there is a decrease in toxins production, more evidence for rifaximin and amoxicillin.

There was equivalence of toxin-like peptides (P) amounts in samples A and samples B_(A+)_, after 7 and 14 days of culturing (where SARS-CoV-2 was expected to be present) but also in samples C, where SARS-CoV-2 was expected to be removed by the centrifugation step. The concentration of toxin-like peptides of neg B samples, derived from faecal matter samples of healthy individuals, remains silent at zero values over 30 days of culture, as shown in Figure 1 Panel M. Toxin-like peptides (P) concentrations values of the experimental repetitions at 7, 14, 21, and 30 days, in the cultures, are represented in Figure 1, Panel G–I, decreased after the addition of some antibiotics (Figure 1–Panel L). In Figure 1 Panel I, it is possible to observe that by culturing bacteria from the faecal samples in which SARS-CoV-2 was removed by a centrifugation step (samples C), comparing it to faecal bacterial cultures in which SARS-CoV-2 was still present (Figure 1 Panel G,H), the semiquantitative concentration spectral counting, calculated in absolute e-value [43], of toxin-like peptides produced by the bacteria remained identical over 30 days to those ones of samples A (Figure 1 Panel G) and samples B_(A+)_ (Figure 1 Panel H). Toxin-like peptides production was influenced by the addition of antibiotics (Figure 1 Panel L). The second integrated finding now is how this toxicological event can be blocked more effectively in vitro with some antibiotics (amoxicillin and rifaximin) than others (Table 1, Figure 1 Panel L and N). This production, in absence of antibiotics, does not end quickly with the removal of the viral pathogen, instead, it continues with a slow decrease over 30 days (Figure 1 Panel M).

## 4. Discussion

Spectral counting is a semi-quantitative evaluation with respect to conditions of absent or low proteins and/or peptides presence [42,43], and it is possible to assess in vitro bacteria cultures, by increasing or decreasing protein concentration under conditions of infection.

It is arguable that these peptides are related to the bacterial Toxin/Antitoxin system [44]. Authors in [45,46] noted how important the function of the respiratory microbiome is and how bacterial peptides can lead to several new mutations in the virus. 

**Figure 2:**
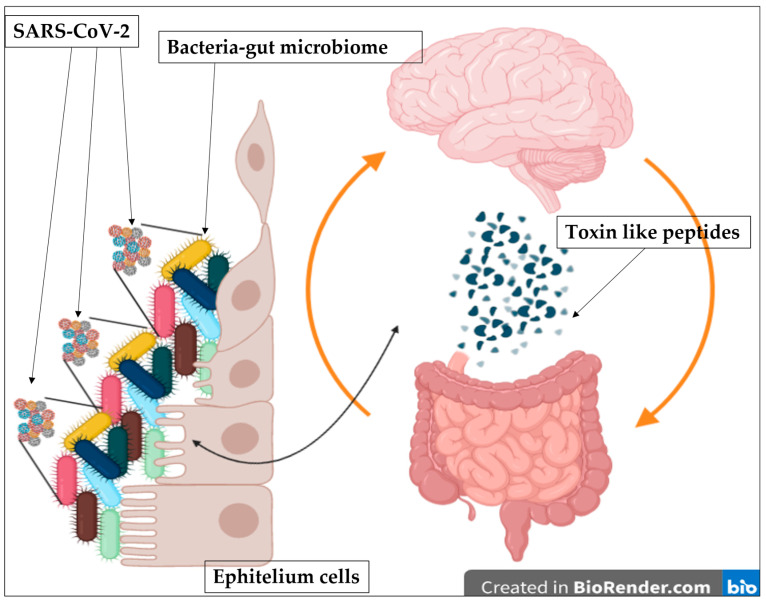
The bacteriophage and toxicological cycle mechanism. Viral infection of the gut microbiome bacteria, activation of toxin production, release of toxin-like proteins into circulation, activation of gut-brain axis, receptor saturation. Image obtained with BioRender.com (28 November 2022).

The gut microbiota act as a defense barrier and help modulate the gut immune system and is essential for gut homeostasis. When it is altered, intestinal disease can occur [47]. An up-regulation or down-regulation of some neuronal genes on iPSC-derived 3D human neural stem cells [33] by using our “toxin-like” peptides derived from bacteria stools samples of COVID-19 patients is also observed. It is discussed that toxins found are similar to conotoxin-like peptides and phospholipase A2, neurotoxins or others (Figure 1 Panels A–F), and it is possible that they can act on the acetylcholine receptors (Figure 2). The unexpected detection of bacterial “toxin-like” peptides that resemble conotoxin proteins was of particular interest. The 4–5 disulfide bridges characterize conotoxins-like proteins, and the cysteine-rich C-CC-C- motif is very similar and is thought to act like the bungarotoxins [48]. Furthermore, the α7 nicotinic receptors’ expression is localized in the glomerular layer of the olfactory bulb (OB) [48,49] and probable interaction of our molecules with this receptor could explain the heterogeneity in the loss and restoration of sense of smell in COVID-19 patients. Authors showed an important perspective on the correlation of nicotinic receptors with the SARS-CoV-2 [14]. Many studies have described how snake venom peptides, might play a role in the loss of smell and taste [50,51,52,53,54]. On the other hand, the toxin-like Phospholipase A2 (PLA2) is also important. PLA2 has been studied for many years; its best-known mechanism of action is on the arachidonic acid inflammatory pathway which increases thromboembolic events [55], with the activating agent thromboxane and from this point of view, it is possible to link to the coagulation disorder found in COVID-19 patients. The data suggested that the toxin-like peptides (P) found might act, because of their heterogeneity and similarity to others already known in nature, on the cholinergic system. Nicotinic and muscarinic receptor subtypes are present in the central nervous system (CNS) on both neurons and glial cells, and they play a crucial role in acetylcholine (ACh)-mediated signalling [56,57,58]. Alterations of this signalling can affect motor control, memory and temperature regulation (hyperthermia that is difficult to control), synapse functions and plasticity, such as auto hetero receptors [57,58,59]. The same is in the peripheral nervous system, where muscle receptors appear to be extensively involved in several processes, such as smooth muscle contraction, glandular secretion, and heart rate regulation [56,57,58]. The observations that toxin-like peptides production was influenced by the addition of antibiotics and that this production does not end with the removal of the viral pathogen are compatible with a model where bacteria are the main producers of these peptides as reaction to SARS-CoV-2 (Figure 1 and Figure 2) which in turn acts for them as an environmental stressor, throughout its bacteriophage-like behavior [58] (Figure 2). Authors in [59] observed that germ-free mice that received microbiome samples from patients with post-COVID-19 syndrome were more susceptible to increased pulmonary problems with *Klebsiella pneumoniae* strain infections and developed cognitive deficits. 

The whole bacterial community needs time to recover from SARS-CoV-2. Probably these peptides are components of a Toxin/Antitoxin bacterial system [26]. The antibiotic rifaximin (rifampicin group) inhibitor bacterial DNA-dependent RNA polymerases and it has antiviral action [60]. It may interact directly with intestinal epithelial barrier cells [61]. Rifaximin and amoxicillin can act on the class of gram-positive and gram-negative bacteria producing peptides [62,63,64]. In the bacteriophage mechanism of the virus infection observed by previously [34,58], the presence of toxins produced by bacteria continuously after viral aggression can be blocked using an appropriate combination of antibiotics in vitro models. 

For the benefit of public health, we must consider that the immuno-compromised elderly population has poor beneficial and immunomodulatory gut microbiota [65], in contrast to a healthy individual with a microbiota represented by 93% Firmicutes and Bacteroidetes [66]. In addition, with aging, facultatively anaerobic bacteria and Gram-negative bacteria, are observed to change in the mucosa due to changes in the living environment, possible malnutrition, and drug intake [66]. This implies an increased susceptibility of the elderly population to viral diseases that have a possible mechanism of microbiota involvement. Considering that bacteria are able, under stressful conditions, such as the presence of a new viral pathogen, to produce oligopeptides or substances that may interfere with the intruder as a likely defense mechanism, suggests more studies aimed at evaluating both the use of probiotics [66] but also oral intake attenuated virus vaccine solutions, probably in order to anticipate the encounter between the virus and the microbiota [59]. However, how the viral pathogen and bacteria may interact or interfere with the host’s various immune and neurobiological mechanisms remains to be elucidated.

## 5. Conclusions

The gut and lung microbiomes appear to play well-defined roles in the mechanisms of viral invasion [67]. The gut microbiota and bacteria could have a key role in virus invasion, and many studies have put evidence of RNA findings in feces [68]. Microbiota divergence is related to socioeconomic background and may correlate with COVID-19 disease severity [69]. In patients with COVID-19 disease, antibiotics are used to treat secondary infections [70,71]. However, overuse of antibiotics is correlated with a reduction in human microbiota beneficial to the host immune system, such as *Eubacterium Rectale, Faecalibacterium Prausnitzii, Ruminococcus Obeum, Dorea Formicigenerans,* and the Lachnospiraceae Family [69]. 

The in vitro evaluation, in bacterial cultures in presence of SARS-CoV-2, of the action of some antibiotics on toxicological aspects needs future investigation and suggests that modulation of the microbiota probably through the use of probiotics. As we reported in the conclusions of our previous work, inducing microbiota resistance against SARS-COV-2 is an option to be considered, probably on a par with what Dr. Sabin did with polio by administering an attenuated oral vaccine over several sessions, it could now be considered as a preventive alternative having to act on both surface immunity and bacteria as we reported [58].

It is not yet possible to define whether we are dealing with a bacterial toxin/antitoxin system or whether it is a mechanism peculiar to the interaction between SARS-CoV-2 and the host microbiota and repeating the same process with other viral RNA pathogens might help to assess the differences or similarities, but certainly, it can be emphasized how important surface immunity is and how much more there is to investigate.

It was hypothesized that this finding in the bacterial dysbiosis argument might suggest some mechanism in continuing long COVID symptoms related to bacterial dysbiosis and their toxicological product should be further investigated.

## Figures and Tables

**Table 1:** Antibiotics are used in bacterial samples. Legend for viral RNA load: + slight increase, ++ marked increase; ---- decrease of viral RNA load 100%; --- decrease of viral RNA load 65–85%, -- decrease of viral RNA load 64–40%, - decrease of viral RNA load 39–25%. Legend for toxin aspect: + Slightly present, ++ moderately present, +++ very present. For more info, see Figure 3 of Petrillo et al. [34].

Drugs	Viral RNA Load	Toxins Aspect
Rifaximin	Decrease -	Not present
Azithromycin	Decrease ----	Present +
Erythromycin	Increase +	Present ++
Metronidazole	Decrease ----	Present ++
Clindamycin	Not change	Present +++
Lincomycin	Increase +	Present +++
Piperacillin + tazobactam	Decrease --	Present +
Vancomycin	Decrease ----	Present +
Amoxicillin	Decrease ----	Present +
Ampicillin	Decrease --	Present +
Cefixime	Decrease ---	Present +
Ceftriaxone	Decrease --	Present +
Meropenem	Decrease -	Present ++
Gentamicin	Decrease -	Present ++
Ciprofloxacin	Decrease --	Present ++
Colistin	Increase +	Present ++
Teicoplanin	Decrease --	Present +
Levofloxacin	Increase ++	Present ++

## Data Availability

Not applicable.
